# Identification of Key Genes and Pathways Associated with *PIEZO1* in Bone-Related Disease Based on Bioinformatics

**DOI:** 10.3390/ijms23095250

**Published:** 2022-05-08

**Authors:** Yuanyuan Zhou, Chen Zhang, Zhongguo Zhou, Chao Zhang, Jiali Wang

**Affiliations:** 1 School of Biomedical Engineering, Sun Yat-sen University, Guangzhou 510006, China; zhangch298@mail2.sysu.edu.cn (C.Z.); zhchao9@mail.sysu.edu.cn (C.Z.); 2 School of Chemistry and Molecular Biosciences, The University of Queensland, Brisbane City 4072, Australia; zhongguo.zhou@uqconnect.edu.au

**Keywords:** bone-related diseases, *Piezo1*, overlapping genes, signaling pathways, bioinformatics

## Abstract

PIEZO1 is a mechano-sensitive ion channel that can sense various forms of mechanical stimuli and convert them into biological signals, affecting bone-related diseases. The present study aimed to identify key genes and signaling pathways in *Piezo1*-regulated bone-related diseases and to explain the potential mechanisms using bioinformatic analysis. The differentially expressed genes (DEGs) in tendon, femur, and humerus bone tissue; cortical bone; and bone-marrow-derived macrophages were identified with the criteria of |log2FC| > 1 and adjusted *p*-value < 0.05 analysis based on a dataset from GSE169261, GSE139121, GSE135282, and GSE133069, respectively, and visualized in a volcano plot. Venn diagram analyses were performed to identify the overlapping DEGs expressed in the above-mentioned tissues. Gene Ontology (GO) enrichment analysis, Kyoto Encyclopedia of Genes and Genomes (KEGG) analysis, protein–protein interaction (PPI) analysis, and module analysis were also conducted. Furthermore, qRT-PCR was performed to validate the above results using primary chondrocytes. As a result, a total of 222 overlapping DEGs and 12 mostly overlapping DEGs were identified. Key *Piezo1*-related genes, such as *Lcn2*, *Dkk3*, *Obscn*, and *Tnnt1*, were identified, and pathways, such as Wnt/β-catenin and PI3k-Akt, were also identified. The present informatic study provides insight, for the first time, into the potential therapeutic targets of *Piezo1*-regulated bone-related diseases

## 1. Introduction

Mechano-sensitive ion channels are a type of ion channel closely related to biomechanical signal transmission. Once discovered, they attracted widespread attention from scholars all over the world [1]. Under abnormal load conditions, such as obesity, trauma, or joint instability, the bone tissue response to mechanical stress stimulation is a complex and precise process [2]. Mechanical stress can activate transmembrane receptors, such as mechanically sensitive ion channels on the surface of bone cells. These receptors can convert extracellular physical signals into biochemical signals and transmit them to bone cells [3]. The PIEZO1 protein is a mechanically sensitive ion channel that can sense different forms of mechanical stimuli and convert them into biological signals to affect physiological processes [4]. In the human body, PIEZO1 is widely distributed in the skin, bladder, kidney, lung, endothelial cells, and bone-related cells [5,6,7]. Bone-related cells, such as chondrocytes, nucleus pulposus cells, and osteoblasts, can sense mechanical stimuli in the extracellular environment and activate signal transduction pathways through mechanically sensitive ion channels, thus, affecting cell proliferation, differentiation, migration, and apoptosis [8,9,10,11].

The load borne by knee cartilage in daily activities is about three-times the body weight. Chondrocytes perceive and respond to mechanical load stimulation to maintain a steady state in the cartilage [12]. The excessive load to joints resulting in uneven stress on joint surface and dysfunction will further lead to cartilage loss and osteophyte formation, leading to or aggravating osteoarthritis and other bone-related diseases [13]. However, the pathogenesis of bone-related diseases is complex and various parts interact with each other. Presently, research involving bone-related diseases treatment has mainly focused on specific parts or symptoms, such as cartilage degradation, osteophyte formation, synovial inflammation, bone loss, or joint pain, but PIEZO1 plays different roles in different parts of bone tissues [14]. For instance, during the development of OA (Osteoarthritis), chondrocytes secrete IL-1α, which enhances the sensitivity of PIEZO1 mechanical transduction and makes chondrocytes more vulnerable to mechanical damage via feedforward mechanisms [13]. It has been reported that PIEZO1 can inhibit the inflammatory response caused by the TLR/NF-κB signaling pathway. PIEZO1 is considered to regulate bone homeostasis through osteoblast–osteoclast crosstalk and plays an important role in bone remodeling, osteoporosis, osteopenia, and spontaneous fractures [15]. Thus, the development of therapeutic drugs regulating a specific target of PIEZO1 may make drug effectiveness more uncertain and cause a wide range of side effects. Collectively, as shown in this study, we believe that it is of great significance to comprehensively analyze the different genes and related signal pathways regulated by *Piezo1* in different bone tissues.

With the rapid development of high-throughput technologies, such as microarray, second-generation sequencing, and single-cell sequencing, bioinformatics analysis has been widely applied to identify key genes and signal pathways in various diseases to provide accurate diagnosis and personalized treatment. For instance, the OA miRNA interactome and its related pathways were elucidated by performing RNA sequencing and differential expression analysis of miRNA and mRNAs in lesioned OA cartilage in humans by comparing it with preserved cartilage from the same patient [16]. *Il11* and *Chadl* were identified as robust OA risk gens and attractive therapeutic targets via RNA sequencing in subchondral bone and cartilage [17]. *En1* was determined as the key gene of bone density and fracture using whole-genome sequencing and verified by an *En1* conditionally knocked-out mouse [18]. The CircRNA “has_circ_0004674” was discovered to be closely related to osteosarcoma chemoresistance using RNA sequencing, which was confirmed by qRT-PCR [19]. However, besides the research on oncology and cardiovascular diseases, there are very few studies on the function of PIEZO1 in bone-related diseases [20,21]. Therefore, in this project, we compared the gene expression profiles regulated by *Piezo1* in tendon, femur, and humerus bone tissue, cortical bone, and bone-marrow-derived macrophages and identified the key genes and signal pathways regulated by *Piezo1*. Furthermore, functional enrichment analysis and protein–protein interaction (PPI) analysis was performed. The most overlapping DEGs were further verified by qRT-PCR in primary chondrocytes of mice. This study may reveal the potential mechanism of *Piezo1* in bone-related diseases, suggesting its upstream and downstream therapeutic targets and providing evidence for the further development of effective treatment for bone-related diseases.

## 2. Materials and Methods

### 2.1. Gene Expression Profiles in Piezo1 Mutant Tissues

The gene expression profiling in tendon, femur and humerus bone tissue, cortical bone, and bone-marrow-derived macrophages was obtained from GEO datasets GSE169261, GSE139121 [22], GSE135282 [15], and GSE133069 [23], respectively. Three wild-type tendon and three *Piezo1*-mutant tendon samples were retrieved from a mouse dataset obtained using the Illumina NextSeq 500 platform (GSE169261). Two wild-type femur and humerus bone tissue samples and two *Piezo1*-knock-out bone tissue samples were retrieved from a mouse dataset obtained with the Ion Torrent S5 platform (GSE139121). Four cortical bone samples of wild-type mice and four cortical bone samples of *Piezo1*-knock-out mice were retrieved from a mouse dataset obtained with the Illumina HiSeq 2500 platform (GSE135282). Five wild-type bone-marrow-derived macrophages samples and six *Piezo1*-knock-out bone-marrow-derived macrophages samples were retrieved from a mouse dataset obtained with the Illumina HiSeq 2500 platform (GSE133069).

### 2.2. Data Pre-Processing

The raw fastq data were obtained from the EBI database (https://www.ebi.ac.uk/, data set GSE169261 was accessed on 20 September 2021; data set GSE139121 was accessed on 6 April 2020; data set GSE135282 was accessed on 7 December 2019; data set GSE133069 was accessed on 21 August 2019) and assessed to detect sequencing failures using FastQC (V 0.11.9), and lower-quality reads were filtered or trimmed using trimmomatic (V 0.39). HISAT2 (V 2.2.1) was applied to map sequencing reads to a population of mice genomes based on the Hierarchical Graph FM index (HGFM) [24]. Sequence reads were assigned to genomic features with featureCounts (V 2.0.1) to obtain a read counts matrix. All pre-processed data were then analyzed by DESeq2 (V 1.34.0) in the R platform (V 4.1.0). To estimate size factors, the DESep2 package offers the median-of-ratios method, which can be descripted as counts divided by sample-specific size factors determined by median ratio of gene counts relative to geometric mean per gene.

### 2.3. Identifying DEGs

The original gene expression profiles were analyzed to identify the upregulated or downregulated DEGs in *Piezo1* mutant tissues, respectively. The criteria for a DEG were |log2FC| > 1 and adjusted *p*-value < 0.05. The results were visualized in volcano plots.

### 2.4. Identification of Overlapping DEGs in Mice Piezo1 Mutant Tissues

A Venn diagram was used to identify the most overlapping DEGs in different tissues including tendon, femur and humerus, cortical bone, and bone-marrow-derived macrophages. Overlapping DEGs were identified when they appeared in at least two of the tissues. All the DEGs were identified by comparing the gene expression profiles between wild-type tissues and *Piezo1* mutant tissues.

### 2.5. Gene Ontology (GO) Enrichment and Kyoto Encyclopedia of Genes and Genomes (KEGG) Pathway Analysis

GO enrichment analysis and KEGG pathway analysis were performed using the R package clusterProfiler (V 4.0.5) with overlapping DEGs. GO enrichment analysis and KEGG pathway analysis were performed with the thresholds of a *p*-value < 0.05.

### 2.6. Construction of the Protein–Protein Interaction Network

The Search Tool for the Retrieval of Interacting Genes/Proteins (STRING) database (https://string-db.org/, accessed on 30 March 2022) was used to construct the PPI network [25]. The overlapping DEGs were mapped to a STRING list to perform multiple proteins search and get a PPI network with interaction scores >0.4. Cytoscape (V 3.9.0) used to visualize the results from the PPI network and perform module analysis. Genes with a connectivity degree ≥10 were identified as hub genes [26].

### 2.7. Module Analysis

Module analysis was performed using the molecular complex detection (MCODE) plugin on the Cytoscape (V 3.9.0) platform. The parameters set to identify enriched functional modules were as follows: Degree Cutoff = 2, Node Score Cutoff = 0.2, K-Core = 2 and Maximum. Depth = 100. Modules with the MCODE score ≥4 were identified as significant modules and were further evaluated for GO enrichment analysis and KEGG pathway analysis with the threshold of a *p*-value < 0.05.

### 2.8. In Vitro Chondrocyte Verification

#### Quantitative Real-Time Polymerase Chain Reaction

To verify the expression of key genes from the forementioned analysis, mice primary chondrocytes were isolated and cultured [27]. Yoda 1 was used to treat chondrocytes to activate the expression of *Piezo1*. The total RNA was extracted from the cells using TRIzol reagent (Invitrogen). cDNA was synthesized using a RevertAid First Strand cDNA Synthesis Kit (TaKaRa). qRT-PCR was conducted to amplify cDNA using an SYBR Premix Ex Tag Kit (TaKaRa) and a 7500 Real-time detection system (Applied Biosystems, Waltham, MA, USA). The primers used in this work were designed by Sangon Biotech Co., Ltd. (Shanghai, China), and the sequences of these primers are listed in Table 1. The relative expression of each gene was normalized to *Gapdh* and presented in a heatmap after normalization (log10 transformation).

## 3. Results

### 3.1. Identification of DEGs in Piezo1 Mutant Tissues

**Figure 1:**
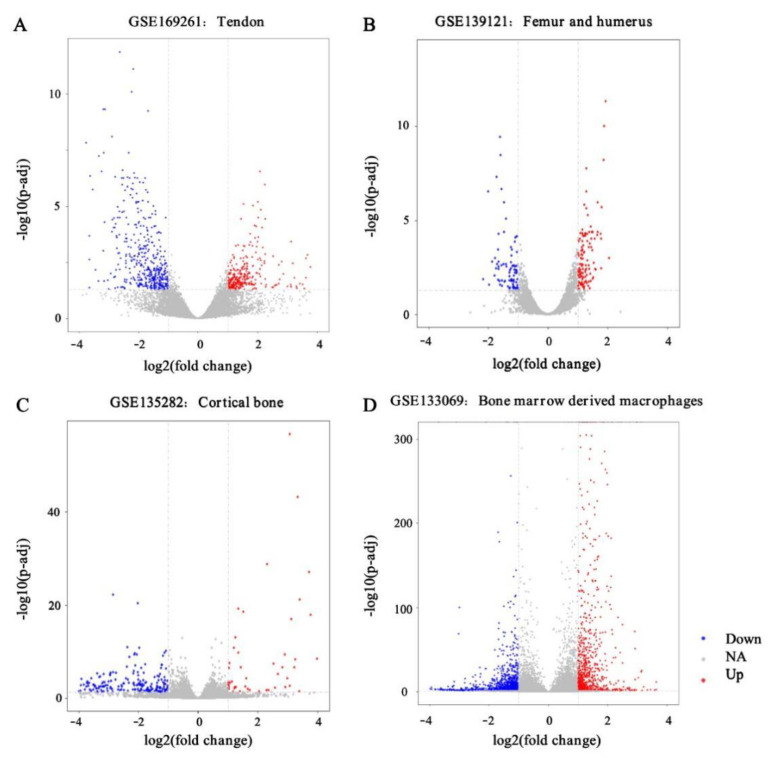
Gene expression profiles of GSE169261 (**A**), GSE139121 (**B**), GSE135282 (**C**), and GSE133069 (**D**) are visualized in volcano plots. DEGs are marked with red, and the criteria for a DEG are |log2FC| > 1.

For the GSE169261 dataset, a total of 766 genes was identified by DESeq2 (V1.32.0), of which 311 were upregulated and 455 were downregulated. For the GSE139121 dataset, 241 genes were identified by DESeq2 (V1.32.0), of which 136 were upregulated and 105 were downregulated. For the GSE135282 dataset, 311 genes were identified by DESeq2(V1.32.0), of which 60 were upregulated and 251 were downregulated. For the GSE133069 dataset, 1653 genes were identified by DESeq2(V1.32.0), of which 767 were upregulated and 886 were downregulated. The distribution of the gene expression for each dataset is visualized in the corresponding volcano plot (Figure 1A–D).

### 3.2. Identification of Overlapping DEGs in Piezo1 Mutant Tissues

**Figure 2:**
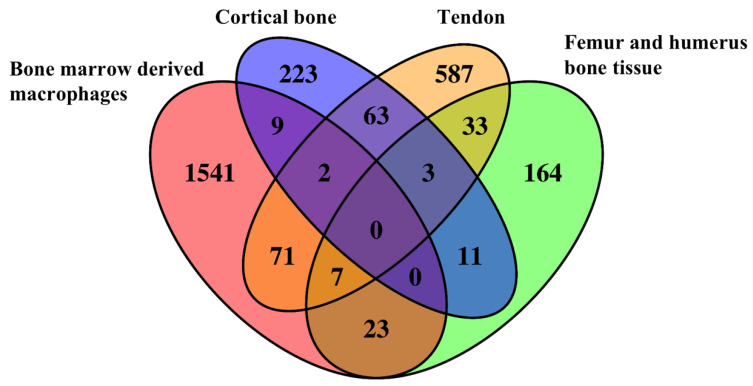
Overlapping DEG distribution in tendon, femur and humerus bone tissue, cortical bone, and bone-marrow-derived macrophages.

As shown in the Venn diagrams, 222 overlapping DEGs were identified (Figure 2), of which the top 30 most regulated genes are listed in Table 2. No overlapping DEGs were found in the tendon, femur and humerus, cortical bone, and bone-marrow-derived macrophages. The genes that appeared the most (i.e., at least three times) were identified as the most overlapping DEGs and are listed in Table 3. A total of 12 of the most overlapping DEGs were identified. Among them, *Lcn2, Creb3l1, Dkk3, Clec5a, Tspan32, Mt1*, and *Hpgd* were differently expressed in bone-marrow-derived macrophages, femur and humerus, and tendon. *Fxyd2* and *Tnnt1* were differently expressed in bone-marrow-derived macrophages, cortical bone, and tendon. *Cox8b, Art1*, and *Obscn* were differently expressed in femur and humerus, cortical bone, and tendon.

### 3.3. GO Enrichment Analysis

**Figure 3:**
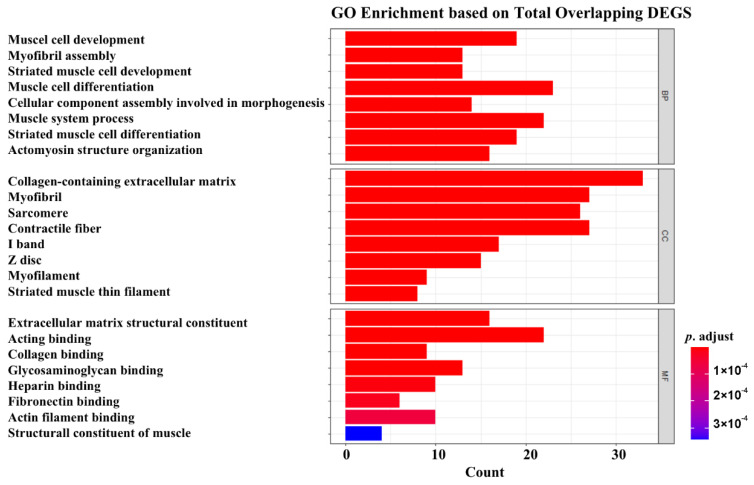
GO enrichment analysis.

The GO enrichment analysis results are presented in Figure 3. The most enriched GO molecular functions were identified as extracellular matrix structure constituents, acting binding, collagen binding, glycosaminoglycan binding, heparin binding, and fibronectin binding. The most enriched GO biological processes mainly included muscle cell development, myofibril assembly, striated muscle cell development, muscle cell differentiation, and cellular component assembly involved in morphogenesis. In addition, the most enriched GO cellular components were collogen-containing extracellular matrix, myofibril, sarcomere, and contractile fiber.

### 3.4. KEGG Pathway Analysis

**Figure 4:**
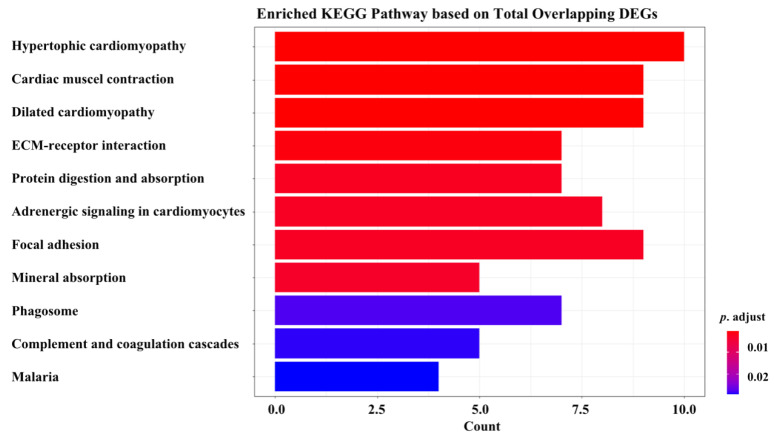
KEGG pathway analysis.

KEGG pathway analysis results are shown in Figure 4, indicating that the total overlapping DEGs were enriched in 11 pathways, including “hypertrophic cardiomyopathy”, “cardiac muscle contraction”, “dilated cardiomyopathy”, “ECM-receptor interaction”, “protein digestion and absorption”, “adrenergic signaling in cardiomyocytes”, “focal adhesion”, “mineral absorption”, “phagosome”, “complement and coagulation cascades”, and “malaria”. Enriched genes located in the corresponding pathways are summarized in Table 4.

### 3.5. PPI Network Analysis

**Figure 5:**
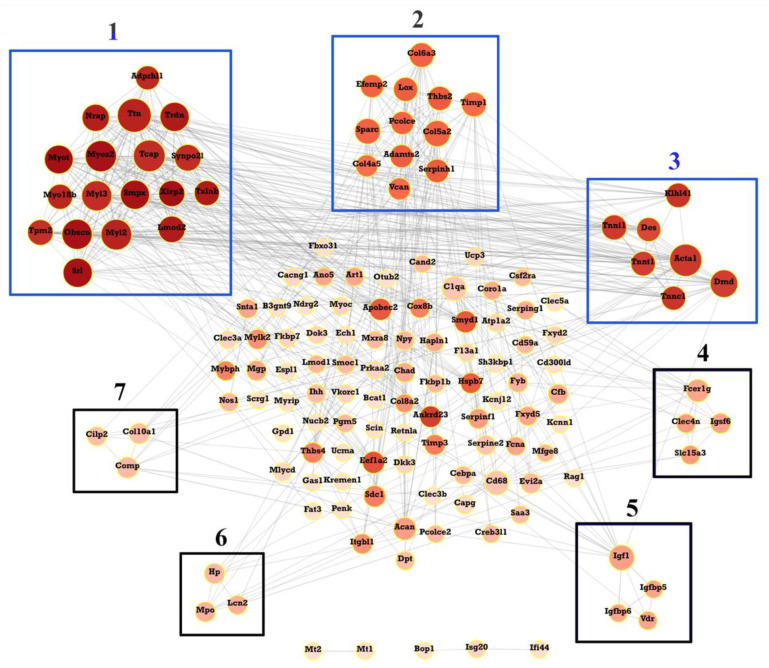
Protein–protein interaction (PPI) analysis. PPI network for all the overlapping DEGs is constructed and followed by module analysis using the MCODE plugin on the Cytoscape (V.3.9.0) platform. Modules with a blue border are significant modules. The size of circles reflects the degree of connectivity.

A total of 578 interactions were obtained with interaction scores > 0.4 using the STRING database. The PPI network was then constructed and presented with the Cytoscape (V.3.9.0) platform (Figure 5). In addition, 47 hub genes were obtained and are presented in Table 5. The top 10 hub genes included *Ttn*, *Acta 1*, *Tcap*, *Myoz2*, *My12*, *Obscn*, *My13*, *Srl*, *Smpx*, and *Trdn*.

### 3.6. Module Analysis

**Figure 6:**
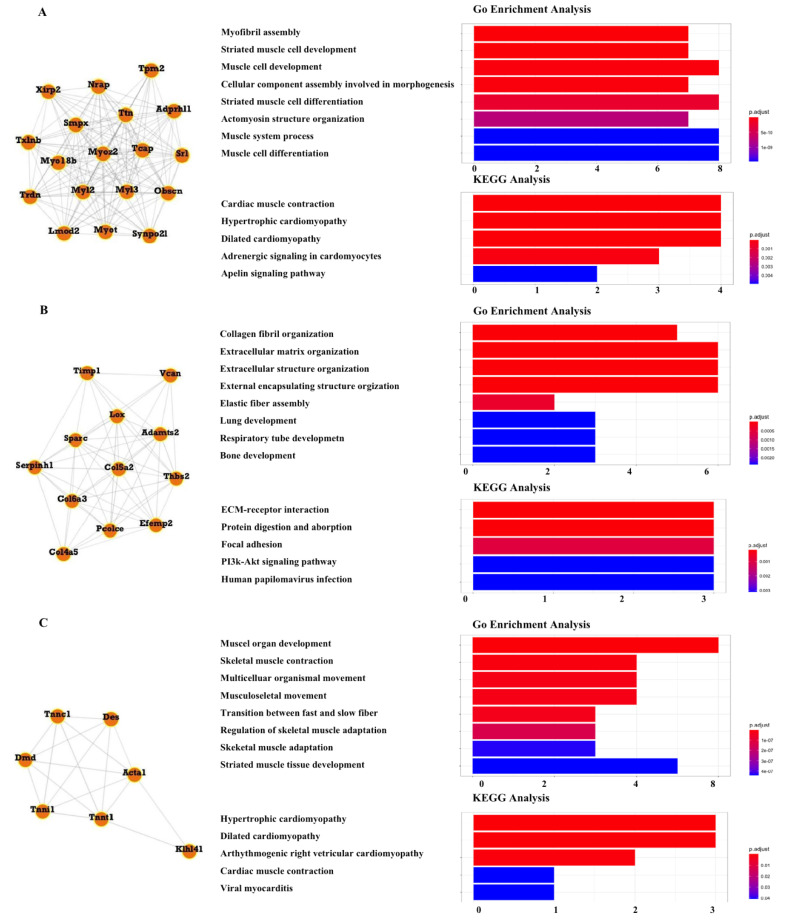
Module analysis. (**A**) Module 1 comprises 18 genes and is enriched in cardiac muscle contraction and hypertrophic cardiomyopathy. (**B**) Module 2 comprises 12 genes and is enriched in ECM–receptor interaction, protein digestion and absorption, and the PI3k-Akt signaling pathway. (**C**) Module 3 comprises 7 genes and is enriched in muscle organ development and skeletal muscle contraction.

A total of seven modules, including three significant modules (Module 1, 2, and 3), were obtained through MCODE analysis (Figure 5). Among the significant modules, Module 1 included 18 genes. GO enrichment analysis showed that Module 1 was enriched in eight functions, such as “myofibril assembly”, “striated muscle cell development”, and “muscle cell development”. For pathway analysis, Module 1 was significantly enriched in various pathways, such as “cardiac muscle contraction”, “hypertrophic cardiomyopathy”, and “dilated cardiomyopathy” (Figure 6A). Module 2 was composed of 12 genes. It was enriched in eight functions, such as “collagen fibril organization”, “extracellular matrix organization”, and “extracellular structure organization”. In addition, “ECM-receptor interaction”, “protein digestion and absorption”, and “PI3k-Akt signaling pathway” were enriched by genes within Module 2 (Figure 6B). Module 3 included seven genes. Enrichment analysis showed that Module 3 was enriched in the “muscle organ development” and “skeletal muscle contraction” functions and “hypertrophic cardiomyopathy” and “dilated cardiomyopathy” pathways (Figure 6C).

### 3.7. In Vitro Chondrocyte Verification

**Figure 7:**
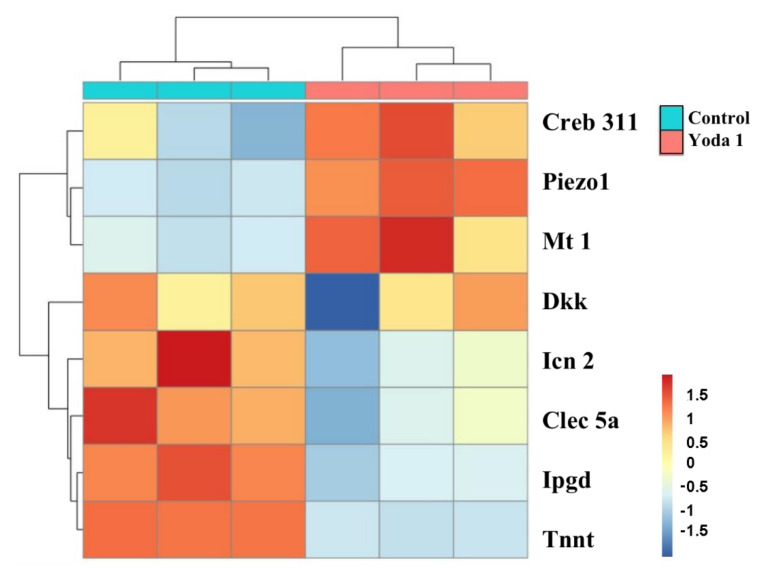
In vitro chondrocytes verification. Heat maps of the relative expression of most overlapping DEGs in mice primary chondrocytes.

To further study the function of *Piezo1* in chondrocytes, Quantitative Real-time PCR assay (qRT-PCR) was performed to evaluate the relative expression of the putative *Piezo1*-related key genes that were identified in the aforementioned results in mice primary chondrocytes. Yoda 1 is a specific agonist of PIEZO1, which affects the sensitivity and inactivation kinetics of mechanically induced responses [28]. Primary chondrocytes of mice were isolated and treated with Yoda 1 (10 μM, 4 h). As a result, *Piezo1* was remarkably overexpressed in Yoda-1-treated chondrocytes. Significantly upregulated *Creb 311* and Mt 1 was observed in *Piezo1*-overexpressed chondrocytes. In addition, *Dkk*, *Icn 2*, *Clec 5a*, *Hpgd*, and *Tnnt* were downregulated in *Piezo1*-overexpressed chondrocytes (Figure 7).

## 4. Discussion

In this study, DEGs were identified in samples of tendon, femur and humerus bone tissue, cortical bone, and bone-marrow-derived macrophages from the GEO database. The key DEGs and their relevant signaling pathways were screened out based on a bioinformatic analysis. This is the first time that overlapping DEGs affected by *Piezo1* in different bone tissues or cells, as well as their enriched functions and pathways, have been analyzed.

We identified a total of 222 overlapping DEGs via Venn diagrams. Those overlapping DEGs that are differentially expressed in at least three tissues or cells were identified as the most overlapping DEGs, including *Lcn2*, *Creb3l1*, *Dkk3*, *Clec5a*, *Tspan32*, *Mt1*, *Hpgd*, *Fxyd2*, *Tnnt1*, *Cox8b*, *Art1*, and *Obscn.* Among all them, *Cox8b*, *Fxyd2*, *Lcn2*, *Obscn*, *Clec5a*, *Dkk3*, *Creb311*, *Tspan32*, and *Tnnt1* were remarkably regulated, with a more than 2-fold change [29]. Moreover, *Tnnt1* is included in our PPI network and plays a key function in Module 3 and Module 1, suggesting their key roles in bone-related diseases. Among those most overlapping DEGs, there is no literature on bone-related diseases associated with *Cox8b*, *Tspan32*, or *Obscn*.

Apart from that, most overlapping DE genes, which were reported to be involved in bone-related diseases, were discussed. *Lcn2* has generally been considered a mechanosensitive gene in bone homeostasis. Mice receiving experimentally induced mechanical unloading exhibited higher bone *Lcn2* expression and bone mass decline [30]. In addition, a significant progressive increase in serum LCN2 levels was observed in healthy subjects undergoing 15 days of bed rest, which induced bone loss [31]. *Lcn2* was reported to negatively regulate the proliferation and differentiation of osteoclast precursors to impair osteoclast formation [32]. Similarly, when osteoclast precursors were preincubated with *Lcn2*, the proliferation and differentiation of osteoclasts were inhibited [32]. As *Piezo1* also showed its modulation function in bone loss and osteophyte formation, the relationship of *Piezo1* and *Lcn2* requires further investigation.

CLEC5A (C-type lectin domain family 5 member A) is a key regulator of synovial injury and bone erosion during autoimmune joint inflammation. The activation of CLEC5A can increase the recruitment of inflammatory macrophages to the joint, promote bone erosion, and enhance the expression of pro-inflammatory cytokines and chemokines in myeloid cells. Moreover, clinical symptoms of autoimmune joint inflammation can be reduced by blocking CLEC5A receptors, suggesting that CLEC5A receptors may be a promising therapeutic target for immune-mediated bone disorders [33,34].

The Wnt/β-catenin signaling pathway plays an important role in inflammatory regulation and cell fate determination, which were studied comprehensively [35,36,37]. As a negative regulator of the Wnt signaling pathway, DKK3 plays an important role in inhibiting cell proliferation or accelerating cell apoptosis [38,39,40]. A recent study of a mouse fracture and repair model showed that DKK3 is expressed in mesenchymal progenitor cells in the periosteum and bone marrow and is a key factor to block osteoblast chondrogenic development [41]. miR-129-5p was found to be able to directly target the DKK3-mediated Wnt/β -catenin pathway, and the miR-129-5p/Dkk3 axis contributes to osteogenesis and bone regeneration, which may provide new therapeutic strategies for maintaining bone stability in bone defects or other bone-related diseases [42].

CREB3L1 is a transcription factor that activates genes involved in the assembly of the collagen-containing extracellular matrix [43]. CREB3L1 termination codon mutations lead to severe osteopenia and reduced amounts of COL1α1 [44], which is usually expressed in connective tissues. The COL1α1 defect has been shown to induce osteogenesis and osteoporosis [45,46]. In an innovative study, CREB3L1-deficient mice developed abnormal bones due to a lack of collagen extracellular matrix produced by osteoblasts [47]. In a clinical study of osteogenesis imperfecta in cat, the CREB3L1 mutation was found to cause severe osteogenesis imperfecta [48].

Tnnt1 is a protein associated with troponin that is critical for the generation of the mechanical forces required for muscle contraction, and loss of Tnnt1 leads to skeletal muscle atrophy [49]. Thus, we presumed that *Tnnt1* and *Piezo1* may be closely related, playing a key role in maintaining bone balance, which deserves deep research.

Mt1 acts as a melatonin receptor and binds to melatonin to prevent oxidative stress, inflammation, and mitochondrial dysfunction to play an anti-aging role. The expression of melatonin and its receptors decreases with age, which can lead to osteoporosis [50]. From the qRT-PCR results of *Piezo1*-upregulated chondrocytes in this project, Mt1 was also remarkably upregulated, which shows a consistent function in osteoporosis and osteosclerosis. In 2008, HPGD was first discovered to be able to lead to autosomal recessive primary hypertrophic osteoarthropathy, which reduces its metabolite levels by causing elevated levels of prostaglandin E2 (PGE2) [51,52]. However, the relationship of these identified genes and Piezo1 are lacking in the research, and changing this could provide useful information for further investigation.

According to KEGG pathway analysis based on overlapping DEGs, apart from cardiac muscle and cardiovascular diseases, general biological function, and malaria, pathways enriched in mineral absorption were shown to play key roles in several bone-related diseases, such as bone loss [53] and osteoporosis [54], suggesting that when it comes to osteoporosis research, it is meaningful to consider mineral absorption by targeting *Piezo1.*

From the results of PPI network and module analysis, a total of 578 interactions, 47 hub genes, and 3 significant modules were obtained. Taking most overlapping DEGs together, *Tnnt1* is a hub gene and located in Module 3, whose function has been discussed before. In Module 2, the PI3k-Akt signaling pathway is closely involved in the inflammatory response to induce MMPs, Adamts, IL-1β, and TNF-α in osteoarthritis [55,56]. However, whether it can be affected by *Piezo1* is still not clear yet, indicating a promising direction when performing osteoarthritis research.

The present study also has some limitations. Although we intended to include as many datasets as possible, only four expression profiles met the criteria, which is far from enough to extend to full and more reliable profiles in order to illustrate the function of Piezo1 and its related genes and signaling pathways. In addition, only primary chondrocytes were used to carry out qRT-PCR validation experiments. The relative expression of our identified genes in other components needs to be further validated. Moreover, the expression of *Pizeo1* and our identified genes and pathways in different bone tissues in a diseased state, such as osteoarthritis, osteoporosis, or osteosclerosis, should also be considered and confirmed.

## 5. Conclusions

In conclusion, this project provides a comprehensive bioinformatics analysis of key genes and signaling pathways with the *Piezo1* mutant in different bone tissues. A total of 12 of the most overlapping genes were identified. Among them, key genes, such as *Lcn2*, *Dkk3*, *Obscn*, and *Tnnt1*, along with their biological function, especially the relationship with *Piezo1*, represent a gap in the research so far. Their downstream mechanisms and specific effects on the different joint components also need to be explored. Signaling pathways, such as the Wnt/β–catenin and PI3k–Akt signaling pathways, have been well investigated, even under clinical trials. However, it is still unclear whether *Piezo1* is involved or even plays a critical function in these pathways. Our study provides insight, for the first time, in the identification of potential therapeutic targets of *Piezo1* mutant bone tissue by comprehensively analyzing them based on bioinformatics.

## Figures and Tables

**Table 1:** List of primers used in qRT-PCR experiments.

Gene	Sequence (5′–3′)	
	Forward	Reverse
*Creb 311*	AGCCTTGTGCTTCGTTCTGGTG	CATCGTAGAACAGTAGGCTTCGG
*Mt 1*	AGGCTCTGGGGTTCATCTCT	GCGTGGTAACCTCAGCTTCT
*Dkk*	GGAGGAAGCTACGCTCAATGAG	TTGCCAGGTTCACCTCAGAGGA
*Icn 2*	ATGTCACCTCCATCCTGGTCAG	GCCACTTGCACATTGTAGCTCTG
*Clec 5a*	GGTTTGGTACGTCAGCCTGGAG	GACACAGTCGAAGTTCTGGTCC
*Ipgd*	AAGCAAAACGGAGGTGAAGGCG	GAGCGTGTGAATCCGATGATGC
*Tnnt*	GAGCAGAGGATGACGCCAAGAA	TTCATCTCCCGACCAGTCTGTC
*Gapdh*	AAATGGTGAAGGTCGGTGTGAAC	AACAATCTCCACTTTGCCACTG

**Table 2:** Top 30 most regulated overlapping genes in at least two tissues.

Gene	Fold Change	Gene	Fold Change	Gene	Fold Change
*Mrln*	6.18	*Dupd1*	4.54	*Mybph*	4.06
*Nctc1*	5.82	*Scrg1*	4.48	*Xirp2*	4.04
*Saa3*	5.37	*Mpo*	4.43	*Ucma*	4.04
*Acta1*	4.94	*Tpsab1*	4.36	*Tcap*	3.95
*Myot*	4.81	*Prr33*	4.34	*Tmem52*	3.93
*Eef1a2*	4.79	*Retnla*	4.15	*Plac8*	3.89
*Mylk2*	4.71	*Clec3a*	4.14	*Otub2*	3.78
*2310002L09Rik*	4.71	*Hapln1*	4.12	*Myoz2*	3.74
*Smpx*	4.59	*Cox8b*	4.12	*Ano5*	3.67
*Trdn*	4.56	*Asb12*	4.10	*Mpz*	3.65

**Table 3:** List of the most overlapping DEGs in *Piezo1* mutant tissues.

Gene	Locations
*Lcn2*	Bone-marrow-derived macrophages, femur and humerus, tendon
*Creb3l1*	Bone-marrow-derived macrophages, femur and humerus, tendon
*Dkk3*	Bone-marrow-derived macrophages, femur and humerus, tendon
*Clec5a*	Bone-marrow-derived macrophages, femur and humerus, tendon
*Tspan32*	Bone-marrow-derived macrophages, femur and humerus, tendon
*Mt1*	Bone-marrow-derived macrophages, femur and humerus, tendon
*Hpgd*	Bone-marrow-derived macrophages, femur and humerus, tendon
*Fxyd2*	Bone-marrow-derived macrophages, cortical bone, tendon
*Tnnt1*	Bone-marrow-derived macrophages, cortical bone, tendon
*Cox8b*	Femur and humerus, cortical bone, tendon
*Art1*	Femur and humerus, cortical bone, tendon
*Obscn*	Femur and humerus, cortical bone, tendon

**Table 4:** Enriched pathway and corresponding genes.

Pathway	Enriched Genes
Hypertrophic cardiomyopathy	*Dmd*, *Des*, *Cacng1*, *Myl2*, *Myl3*, *Tnnc1*, *Ttn*, *Igf1*, *Prkaa2*, *Tpm2*
Cardiac muscle contraction	*Cox8b*, *Fxyd2*, *Cacng1*, *Atp1a2*, *Myl2*, *Myl3*, *Tnnc1*, *Trdn*, *Tpm2*
Dilated cardiomyopathy	*Dmd*, *Des*, *Cacng1*, *Myl2*, *Myl3*, *Tnnc1*, *Ttn*, *Igf1*, *Tpm2*
ECM-receptor interaction	*Col6a3*, *Comp*, *Chad*, *Thbs4*, *Sdc1*, *Col4a5*, *Thbs2*
Protein digestion and absorption	*Col6a3*, *Col10a1*, *Col8a2*, *Fxyd2*, *Atp1a2*, *Col5a2*, *Col4a5*
Adrenergic signaling in cardiomyocytes	*Creb3l1*, *Fxyd2*, *Cacng1*, *Atp1a2*, *Myl2*, *Myl3*, *Tnnc1*, *Tpm2*
Focal adhesion	*Col6a3*, *Comp*, *Chad*, *Mylk2*, *Myl2*, *Thbs4*, *Igf1*, *Col4a5*, *Thbs2*
Mineral absorption	*Mt1*, *Vdr*, *Fxyd2*, *Atp1a2*, *Mt2*
Phagosome	*Mpo*, *Mrc2*, *Comp*, *Thbs4*, *Nos1*, *Coro1a*, *Thbs2*
Complement and coagulation cascades	*Cd59a*, *C1qa*, *F13a1*, *Serping1*, *Cfb*

**Table 5:** List of hub genes and connectivity degree.

Gene	Degree	Gene	Degree	Gene	Degree	Gene	Degree
*Ttn*	34	*Nrap*	21	*Klhl41*	16	*Efemp2*	13
*Acta1*	31	*Synpo2l*	20	*Col6a3*	16	*Pcolce*	13
*Tcap*	30	*Lmod2*	20	*Timp1*	16	*Fcer1g*	12
*Myoz2*	29	*Col5a2*	19	*Lox*	15	*Acan*	12
*Myl2*	28	*Xirp2*	19	*Des*	15	*Apobec2*	11
*Obscn*	27	*Tpm2*	18	*Thbs2*	15	*Smyd1*	11
*Myl3*	27	*Sparc*	18	*Adprhl1*	15	*Eef1a2*	11
*Srl*	25	*Txlnb*	17	*Cd68*	15	*Vcan*	11
*Smpx*	25	*Myo18b*	17	*Tnnc1*	15	*Col4a5*	11
*Trdn*	25	*Igf1*	17	*C1qa*	15	*Sdc1*	10
*Myot*	24	*Tnni1*	17	*Serpinh1*	14	*Ankrd23*	10
*Dmd*	22	*Tnnt1*	16	*Adamts2*	13		

## Data Availability

The original contributions presented in the study are included in the article; further inquiries can be directed to the corresponding authors.
